# Outbreak of Sexually Transmitted Nongroupable *Neisseria meningitidis*–Associated Urethritis, Vietnam

**DOI:** 10.3201/eid2910.221596

**Published:** 2023-10

**Authors:** Hao Trong Nguyen, Thanh V. Phan, Hau Phuc Tran, Thao Thi Phuong Vu, Nhi Thi Uyen Pham, Tho Thi Thanh Nguyen, Ha Manh Bui, Bao Hac Duong, Thu Nguyen Anh Luu, Nguyen Nhat Pham, Phuc Duy Nguyen, Tu Ngoc Le, Thu Quang Le, Dai Thi Trang Vo, Lan Trong Phan, Nghia Van Khuu, Quang Duy Pham, Thuong Vu Nguyen

**Affiliations:** Ho Chi Minh City Hospital of Dermato-Venereology, Ho Chi Minh City, Vietnam (H.T. Nguyen, T.T.P. Vu, N.T.U. Pham, T.T.T. Nguyen, H.M. Bui, B.H. Duong, T.N.A. Luu, N.N. Pham);; Pasteur Institute of Ho Chi Minh City, Ho Chi Minh City (T.V. Phan, H.P. Tran, P.D. Nguyen, T.N. Le, T.Q. Le, D.T.T. Vo, L.T. Phan, N.V. Khuu, Q.D. Pham, T.V. Nguyen)

**Keywords:** Neisseria meningitidis, bacteria, sexually transmitted infections, antimicrobial resistance, urethritis, men who have sex with men, Vietnam

## Abstract

We report on an outbreak of nongroupable *Neisseria meningitidis*–associated urethritis, primarily among men who have sex with men in southern Vietnam. Nearly 50% of *N. meningitidis* isolates were resistant to ciprofloxacin. This emerging pathogen should be considered in the differential diagnosis and management of urethritis.

Urogenital and anorectal infections caused by *Neisseria meningitidis* have been reported in several countries and found to be more prevalent among men who have sex with men (MSM) than among heterosexual men or women (*1*–*3*). During 2013–2016, rising numbers of a novel clade of nongroupable *N. meningitidis* (NmNG) urethritis were reported in multiple US cities and have been termed US NmNG urethritis clade (*4*). Two cases of US NmNG urethritis were also documented among MSM in the United Kingdom in 2019 (*5*). We report an outbreak of urethritis associated with US NmNG urethritis clade among men in southern Vietnam.

## The Study

We conducted a matched case–control study to investigate *N. meningitidis* urethritis and risk factors in men seeking treatment for urinary discharge at Ho Chi Minh City Hospital of Dermato-Venereology (HHDV; Ho Chi Minh City, Vietnam). Cases of *N. meningitidis* urethritis were confirmed by either real-time PCR or culture of urethral discharge. Controls were matched to case-patients by age range and sexual orientation (Appendix). During September 2019–December 2020, we recruited 19 case-patients and 76 controls from HHDV (Appendix Figure 1). We collected information on sociodemographic factors, sexual behaviors, and medical history by face-to-face interviews and from medical records (Appendix). The HHDV institutional review board approved the study.

We identified *N. meningitidis* by using bacterial culture and real-time PCR targeting the *sodC* gene (*6*) and determined serogroups by using latex agglutination and real-time PCR. We performed antimicrobial susceptibility testing according to Clinical and Laboratory Standards Institute guidelines (*7*). We conducted whole-genome sequencing, then analyzed multilocus sequence types (MLST) in PubMLST (https://pubmlst.org/organisms/neisseria-spp). We used BEAST (http://beast.community) to estimate the time of bacterial arrival in Vietnam and to conduct antimicrobial-resistance typing (Appendix). We performed conditional logistic regression to assess risk factors for US NmNG urethritis clade by using Stata 14 (StataCorp LLC, https://www.stata.com). We used a log likelihood-ratio test to select the best-fitting model (Appendix).

The mean age of case-patients was lower than that of controls (26.9 vs. 27.8 years). Condom use was low in both case-patients and controls before pyuria developed (5.3% of case-patients, 2.7% of controls). More case-patients than controls lived with male partners (42.1% vs. 13.7%) and had sex with foreign-born persons (15.8% vs. 1.3%). Multivariate analysis results showed that persons living with male partners (adjusted odds ratio [aOR] 14.41, 95% CI 1.01–204.62) and having sex with foreign-born persons (aOR 26.78, 95% CI 1.03–697.82) were more likely to contract US NmNG urethritis (Table 1). Moreover, most (79%) case-patients reported sex with male partners. Persons having oral or vaginal sex with female partners in the past 12 months were less likely to have US NmNG urethritis (aOR 0.13, 95% CI 0.02–0.87). Those findings suggest that the US NmNG outbreak was concentrated within the MSM population.

**Table 1:** Correlates of several selected factors among male patients with *Neisseria meningitidis* US NmNG urethritis and controls, Vietnam*

Variables	Cases, n = 19	Controls, n = 76	Univariable analysis†			Multivariable analysis†	
			OR (95% CI)	p value		aOR (95% CI)	p value
Mean age, y	26.9	27.8	0.90 (0.75–1.07)	0.232		NA	
Mean years of education	10.8	11.0	0.96 (0.75–1.23)	0.760			
Currently living in Ho Chi Minh City	14 (73.4)	56 (73.7)	1.00 (0.30–3.28)	>0.999			
Living arrangements							
Live with a female partner	1 (5.3)	22 (30.1)	Referent			Referent	
Live with a male partner	8 (42.1)	10 (13.7)	17.03 (1.83–158.26)	0.013		14.41 (1.01–204.62)	0.049
Other, e.g., live alone, or with friends or family	3 (15.8)	12 (16.4)	5.44 (0.65–45.54)	0.118		7.0 (0.61–80.24)	0.118
Ever had sex with							
Male sexual partners	14 (73.7)	49 (64.5)	Referent				
Female sexual partners	4 (21.1)	16 (21.1)	0.41 (0.02–7.54)	0.551			
Both male and female partners	1 (5.3)	11 (14.5)	0.17 (0.01–3.17)	0.238			
Ever had oral sex	19 (100.0)	74 (97.4)	NA				
Ever participated in group sex	1 (5.3)	2 (2.6)	2 (0.18–22.06)	0.571			
During past 12 mo							
Oral or vaginal sex with female partner	4 (21.1)	44 (57.9)	0.10 (0.02–0.47)	0.004		0.13 (0.02–0.87)	0.035
Oral or anal sex with male partner	15 (78.9)	39 (51.3)	10.57 (1.28–87.34)	0.029		NA	
Any casual partners	5 (26.3)	34 (44.7)	0.39 (0.12–1.32)	0.131		NA	
Commercial sex worker partner	3 (15.8)	28 (36.8)	0.30 (0.08–1.15)	0.078			
Drunkenness during sex	3 (15.8)	23 (30.3)	0.33 (0.07–1.65)	0.177			
Sex with a foreign-born partner in the past month	3 (15.8)	1 (1.3)	12.0 (1.25–115.36)	0.031		26.78 (1.03–697.82)	0.048
Condom use during sex before symptom onset‡	1 (5.3)	2 (2.7)	1.81 (0.16–20.08)	0.628			
Used social media sites to find sexual partners	13 (68.4)	32 (42.1)	4.07 (1.17–14.13)	0.027		NA	
Ever used ATS§	1 (5.3)	4 (5.3)	1.00 (0.10–10.07)	>0.999			

*Values are no. (%) except as indicated. ATS, amphetamine-type stimulants; aOR, adjusted odds ratio; NA, not applicable; OR, odds ratio. †Conditional logistic regression. ‡Symptoms were pyuria, dysuria, or both. §Ecstasy, crystal meth (methamphetamine), or other substances used to increase excitement when having sex (also called chemsex).

Among 19 case-patients, 7 were co-infected with >1 other pathogen: 2 (11%) cases of syphilis, 4 (21%) cases of chlamydia, and 1 (0.1%) case involving both ureaplasma and mycoplasma. Ten (83%) case-patients without co-infections and 6 (86%) with co-infections experienced >1 symptom. Pyuria was reported in 2 (29%) co-infected case-patients, and dysuria was reported in 10 (83%) case-patients without co-infections and 3 (43%) with co-infections (Appendix Table 1). Most participants had not received meningococcal vaccines, nor recalled being vaccinated against *N. meningitidis* (Table 2). The prevalence of HIV, syphilis, and chlamydia infections was higher among case-patients compared to controls but not statistically significant, whereas gonorrhea was only found in controls (98.7%) (Table 2). Multivariate analysis showed that those who had US NmNG urethritis were less likely to report burning sensations during urination (odds ratio [OR] 0.08, 95% CI 0.01–0.46) and more likely to delay seeking treatment (OR 16.0, 95% CI 2.0–127.54) (Table 2). 

**Table 2:** Correlates of demographic characteristics, STI symptoms and pathogens among male patients with *N. meningitidis* US NmNG urethritis and controls, Vietnam*

Variables	Cases, n = 19	Controls, n = 76	Univariable analysis†			Multivariable analysis†	
			OR (95% CI)	p value		aOR (95% CI)	p value
Mean age, y	26.9	27.8	0.90 (0.75–1.07)	0.232		NA	
Mean years of education	10.8	11.0	0.96 (0.75–1.23)	0.760		NA	
Medical examination >3 d after symptom onset	13 (68.4)	11 (14.5)	18.41 (4.04–83.86)	<0.001		16.00 (2.00–127.54)	0.009
Symptoms							
Pyuria	2 (10.5)	10 (13.2)	0.79 (0.16–3.79)	0.764		NA	
Dysuria	13 (68.4)	65 (85.5)	0.39 (0.12–1.19)	0.098		NA	
Burning sensation during urination	2 (10.5)	57 (75.0)	0.05 (0.01–0.22)	<0.001		0.08 (0.01–0.46)	0.005
Discharge	4 (21.1)	16 (21.1)	1.00 (0.26–3.89)	>0.999			
Time between symptom onset and medical consultation, d							
Mean	4.7	3.0	1.92 (1.18–3.13)	0.009		NA	
Median (range)	5 (3–12)	3 (1–14)					
History of meningococcal vaccine							
Ever	0	1 (1.3)	NA				
Never	12 (63.2)	51 (67.1)	NA				
Do not know, do not remember	7 (36.8)	24 (31.6)	NA				
Positive tests							
HIV	2 (10.5)	6 (7.9)	1.36 (0.26–7.20)	0.716		NA	
Syphilis	2 (10.5)	3 (3.9)	2.67 (0.45–15.96)	0.283		NA	
Gonorrhea	0 (0.0)	75 (98.7)	NA			NA	
Chlamydia	4 (21.1)	7 (9.2)	2.57 (0.67–9.83)	0.168		3.83 (0.37–39.43)	0.258
Ureaplasma	1 (5.3)	8 (10.5)	0.48 (0.06–4.01)	0.502		NA	
Mycoplasma	1 (5.3)	10 (13.2)	0.39 (0.04–3.10)	0.372		NA	

*Values are no. (%) except as indicated. aOR, adjusted odds ratio; NA, not applicable; OR, odds ratio. †Conditional logistic regression.

In this study, uncomplicated gonorrhea was treated with a single 500-mg intramuscular dose of ceftriaxone, followed by either a 7-day course of doxycycline (100 mg 2×/day) or a single 1,000-mg dose of azithromycin. In Vietnam, gonococci isolated in 2011 and during 2015–2016 increasingly resisted antimicrobial drugs except for ceftriaxone, spectinomycin, and azithromycin (*8*). Moreover, 98.3% of *N. gonorrhoeae* isolates were ciprofloxacin-resistant (*9*). In a national survey conducted in Vietnam, 30% of persons reported purchasing antibiotics primarily for addressing symptoms, including genitourinary manifestations, and 81.7% did so without a prescription; ciprofloxacin was among the top 5 antimicrobial drugs acquired (*10*). In another study among MSM in Vietnam, 64% reported ever taking antibiotics without a prescription (*11*).

**Figure 1:**
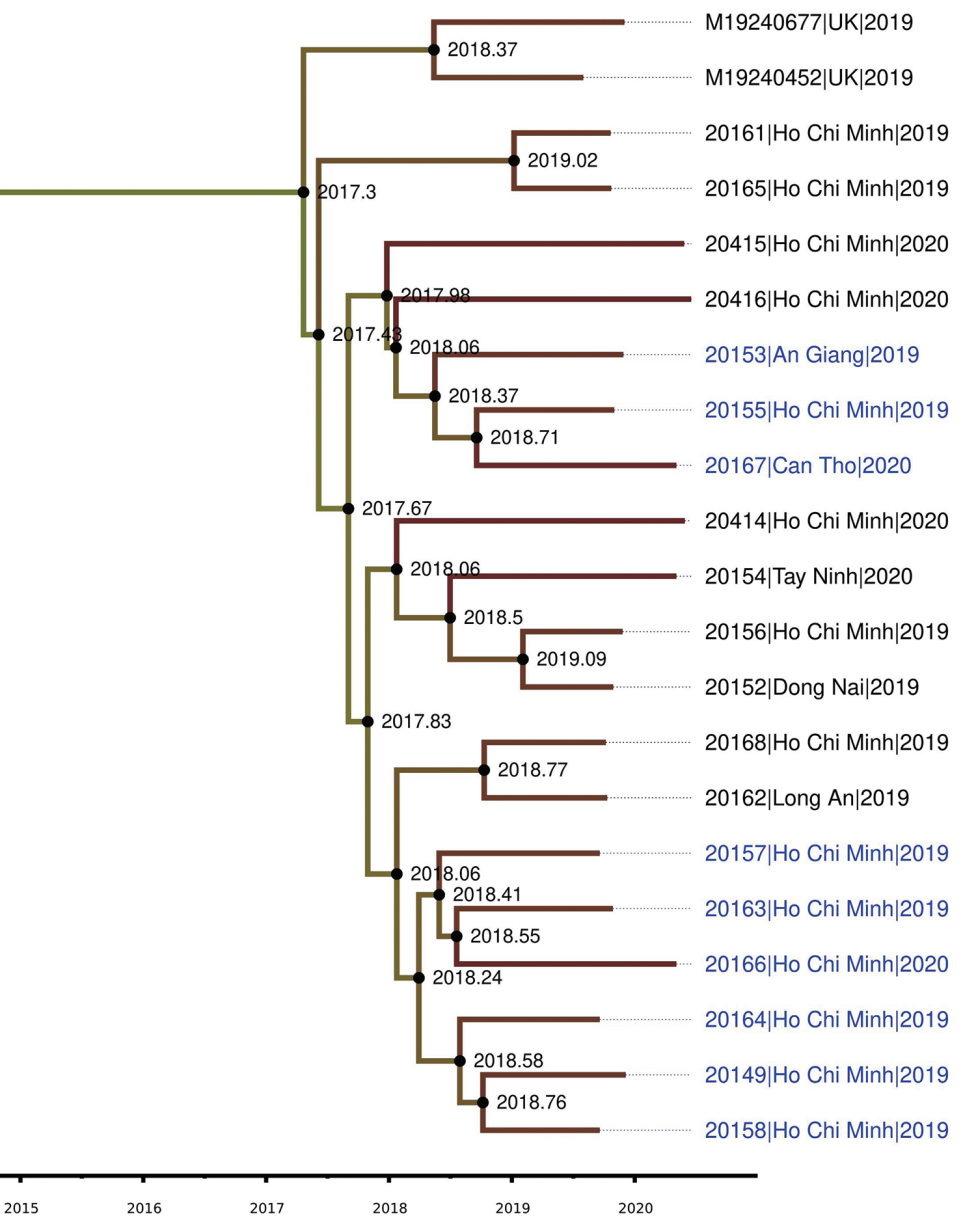
Plylogenetic tree of isolates from an outbreak of sexually transmitted nongroupable *Neisseria meningitidis*–associated urethritis, Vietnam. Phylogenetic tree was constructed using Baysian Skygrid model, performing with BEAST/BEAGLE version 1.10.4 (https://beast.community/beagle), and displaying with FigTree version 1.4.4 (http://tree.bio.ed.ac.uk/software/Figtree). Blue text indicates ciprofloxacin-resistant strains. Scale bar indicates the time of evolutionary history.

The US NmNG urethritis clade in our study displayed intermediate susceptibility to penicillin, with MIC values ranging from 0.125–0.38 mg/L. Nine of the 19 isolates demonstrated resistance to ciprofloxacin (MIC 0.19–3.0 µg/mL). MLST analysis revealed that the isolates belonged to the sequence type 11 complex (Appendix Table 2). A phylogenetic tree displayed the isolates from Vietnam and the United Kingdom forming a monophyletic clade with those from Ohio, USA, one of the 2 US NmNG urethritis clades (*12*) (Appendix Figure 2). BEAST analysis estimated that the time of most recent common ancestor of Vietnam and UK isolates appeared between 2016 and 2018 (median 2017.3; 95% high posterior density interval 2016.4–2018.1), with a Bayesian posterior probability of 1.0 (Figure 1; Appendix Figure 3).

**Figure 2:**
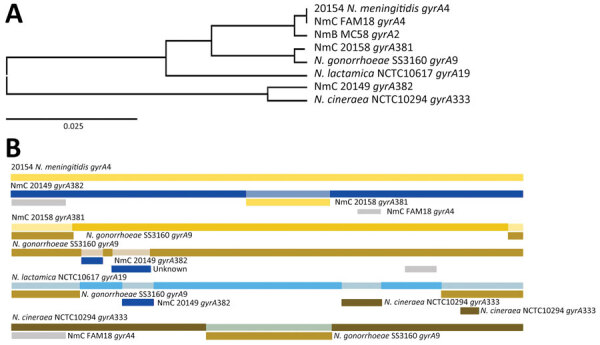
Whole-genome analysis of isolates from an outbreak of sexually transmitted nongroupable *Neisseria meningitidis*–associated urethritis, Vietnam. Comparison generated in RDP4 (https://rdp4.software.informer.com) for full length of 2,751-bp. A) Tree shows the genetic relationship between isolate 20158_*gyrA381* and gonococci_*gyrA9* using unweighted pair group method with arithmetic mean of the region derived from their parents, beginning at 1 to ending breakpoint at 337 bp. B) Bars show potential recombination breakage points identified with at least 1 of the 7 methods contained in RDP4. NmB, *N. meningitidis* B; NmC, *N. meningitidis* C.

All isolates carried the penA_316 allele and point mutations that reduced their susceptibility to penicillin (*13*). Nine ciprofloxacin-resistant isolates exhibited 2 new alleles in *gyrA*, assigned as *gyrA*_381 (n = 8), which had dual mutations at T91F and D95A, and *gyrA*_382, which had monomutation at T91I. We used RDP4 (https://rdp4.software.informer.com) to analyze the full 2,751-bp length and found that *gyrA*_381 received a fragment containing a mutation from gonococci (Figure 2). Our study revealed that isolates containing mutations at both T91F and D95A in the *gyrA* gene displayed a high level of resistance to ciprofloxacin, similar to that found in *N. gonorrhoeae* (*14*). Moreover, isolate 20158 had a mutation at S87R of *parC* and the *gyrA*_381 allele had an elevated MIC of 3 µg/mL.

The emergence of ciprofloxacin-resistant *N. meningitidis* US NmNG urethritis clade in Vietnam is a major concern, especially considering ciprofloxacin resistance is rare in the United Kingdom and United States (*4*,*5*). A previous study observed that strains with MICs >0.064 mg/L were correlated with alterations in *gyrA* (*15*). Hence, when specimens cannot be cultured, *gyrA*-sequencing can be particularly useful in predicting susceptibility of ciproloxacin.

## Conclusions

We report an outbreak of US NmNG urethritis among men in Vietnam, predominantly MSM. Having sex with foreign-born persons and living with male partners were factors strongly associated with the disease. Isolates in this outbreak might have originated from the Ohio (USA) clade and were mainly resistant to ciprofloxacin, which is commonly used for prophylaxis against invasive meningococcal diseases in Vietnam. 

Symptoms among patients with US NmNG urethritis were milder than those in controls with gonococcal urethritis. Because US NmNG urethritis is less likely than gonococcal urethritis to manifest symptoms, clinicians should consider *N. meningitidis* when managing patients with urethral discharge. Bacterial culture should be routinely performed on urethritis specimens that test *N. gonorrhoeae*–negative by nucleic acid amplification tests to determine whether the infection is caused by *N. meningitidis*. More studies with larger sample sizes should be conducted to provide a more comprehensive picture of the burden and clinical symptoms of US NmNG urethritis. 

In conclusion, our findings emphasize the importance of ongoing monitoring of appropriate use of antibiotics and antimicrobial resistance to prevent the further spread of resistant US NmNG urethritis clade. Thus, clinicians should be aware of this emerging bacterium and include US NmNG in the differential diagnosis for urethitis.

Appendix Additional information on an outbreak of sexually transmitted nongroupable *Neisseria meningitidis*–associated urethritis, Vietnam.
