# Supplementary material for: Outbreak of Sexually Transmitted Nongroupable Neisseria meningitidis–Associated Urethritis, Vietnam
Source: Emerg Infect Dis. 2023 Oct;29(10):2130–4. doi: 10.3201/eid2910.221596 (PMC10521605; doi:10.3201/eid2910.221596)
Supplement: Appendix — Additional information on an outbreak of sexually transmitted nongroupable Neisseria meningitidis–associated urethritis, Vietnam. [file 22-1596-Techapp-s1.pdf]

Article DOI: https://doi.org/10.3201/eid2910.221596

EID cannot ensure accessibility for supplementary materials supplied by authors. Readers who have difficulty accessing supplementary content should contact the authors for assistance.

Outbreak of Sexually Transmitted Nongroupable *Neisseria meningitidis*–Associated Urethritis, Vietnam

Appendix

Methods

A matched case-control study was conducted among male patients with urinary discharge seeking treatment at Ho Chi Minh City Hospital of Dermato-Venereology (HHDV). A case was defined as a man who sought sexually transmitted infection (STI) treatment and care at HHDV and presented with urinary discharge and was confirmed with either real-time PCR or culture of urethral discharge specimen. A control was defined as a man who visited HHDV with the presence of urinary discharge with a diagnosis of a non–*Nessaria meningitidis* infection. For each case, four controls were recruited, matching the case-patient by age ranges (16–19, 20–29, 30–39, and ≥40 years) and sexual orientation (having sex with females only, having sex with males). In total, there were 19 cases and 76 controls recruited to the study during 9/2019 to 12/2020. From September 2019 to December 2020, 2,702 male patients were diagnosed with urethritis. Most male patients presented with symptoms of pyuria, dysuria, burning sensation during urination, and dripping. The study was approved by the HHDV institutional review board.

Data Collection

Demographic and other information of the cases, including age, gender, place of residence, clinical symptoms, diagnosis, and treatment, were retrieved from medical records and screening tests for HIV, chlamydia, syphilis, gonorrhea, ureaplasma, and mycoplasma that had been performed at the HHDV. Investigators conducted face-to-face interviews with the cases using a standardized questionnaire. Patients were asked about their social demographic

characteristics, medical and immunization history, access to social networks, and sexual and drug use behaviors. The controls were also interviewed using the same questionnaire.

Laboratory Investigation

Isolate Collection

At the HHDV, men presenting with urinary discharge were initially suspected to have gonococcal urethritis. However, 19 of the collected specimens tested negative for *Gonococci* using real-time PCR and subsequent bacterial culture yielded 19 isolates of Gram-negative diplococci, which could not be identified as gonococci. These isolates were then transferred to Pasteur Institute of Ho Chi Minh for further laboratory examination to confirm the actual etiology.

Identification and Characterization of *N. meningitidis*

To identify *N. meningitidis*, two methods were used: 1) a biochemical test kit of Analytical Profile Index Neisseria-Haemophilus (API-NH, bioMérieux); and 2) species-specific superoxide dismutase gene (¬sodC) real-time PCR (Quanta Bioscience). A slide agglutination serogrouping (SASG) kit of N. meningitidis Antiserum (Difco) and serogroup-specific (cps) gene rt-PCR were used to determine the serogroup of the samples, among A, B, C, W, X, Y or nongroupable (NG).

Antimicrobial Susceptibility Testing

Antimicrobial susceptibilities for the urethral isolates were determined through MIC with E-test, using six antibiotics: penicillin, cefotaxime, meropenem, azithromycin, ciprofloxacin, and rifampin (Liofilchem). The Clinical and Laboratory Standard Institute (CLSI) 2017 was used to determine breakpoints for susceptible, intermediate, or non-susceptible/resistant (*1*).

Whole-Genome Sequencing

Whole-genome sequencing of 19 urethral *N. meningitidis* isolates was performed at the PIHCM, Vietnam, from which DNA was extracted using the QIAmp DNA mini kit (QIAGEN). Libraries were then constructed using the Nextera XT DNA Library Prep Kit v2.0 (Illumina). Next, an Illumina-Miseq Platform generated 300-bp paired-end reads by running 300 cycles. Fastq reads were trimmed with Trimmomatic, and de novo assembled with SPAdes v3.13 (*2*,*3*). Multilocus sequence typing (MLST), fine-typing antigens, targeted outer-membrane proteins

(OMV), and antibiotic resistance genes were obtained through BIGSdb (*4*). All draft genomes were submitted to the National Center for Biotechnology Information (NCBI) under BioProject no. PRJNA672782 and were also uploaded to the PubMLST database.

Phylogenetic Analysis

Phylogenetic inferences based on differences in the gene-by-gene comparison of 1,605 defined loci of the core-genome MLST were made. The genomes of the isolates in Vietnam were compared against those in the United States (n = 209) and United Kingdom (n = 2), using an invasive isolate, M21273, as a reference (*5*,*6*). The phylogeny was inferred using the PubMLST-based Genome Comparator tool.

A subset of 81 genomes of the US NmNG UC isolates and 7 of invasive CC-11 as an outgroup was inferred with a time-measure phylogeny with BEAST v1.10 (*7*). A concatenated core alignment of 1,275,571 bps was generated through the genome-comparator and aligned with MAFFT. Subsequently, it was masked with Gubbins v2.2.1, leaving 1,081 bp of polymorphic sites (*8*). The Gubbins-masked alignment was generated using maximum-likelihood phylogeny with IQ-Tree, replicating 1,000 ultrafast-bootstrap (*9*).

To examine temporal signals, a root-to-tip linear regression was constructed with TempEst v1.5.3, estimating correlation coefficient was 0.91 and R^2 was 0.83 (*10*). A time-measured phylogeny was inferred by BEAST/BEAGLE, using the general time-reversible (GTR) substitution model and gamma heterogeneity site. The uncorrelated exponential relaxed clock (UCED), which allows each branch to evolve independently, was selected over the other molecular clocks, such as relaxed uncorrelated lognormal (UCLN), random local clock (RLC), and strict clock. The model was selected by using generalized stepping-stone sampling and estimation of the most recent common ancestor (TMRCA) of the subset. The marginal likelihood estimation was also used to evaluate the flexible nonparametric Skygrid model and other parametric models: constant, expansion, logistic, exponential growth, and GMRF Skyride. The Bayesian Skygrid was performed with 87 parameters corresponding to 88 sequences, and 17 grid points as the length of time from the newest to the root of the tree, running 250 million steps for two separate times.

Testing for Other Sexually Transmitted Infections (STIs)

Urethral swab specimens were collected following the hospital routine protocol to test for gonorrhea, *Chlamydia*, mycoplasma, and ureaplasma. From each participant 3 mL of venous blood was drawn to test for HIV and syphilis.

HIV was screened using a rapid test (Determine HIV-1/2, Alere Medical Co., Matsudo, Japan) at HHDV. Positive specimens were sent to Center for Disease Control of Ho Chi Minh City to confirm positivity with three different tests: Advia Centaur HIV Ag/Ab Combo (CHIV) Assay, Siemens Healthcare Diagnostics Inc., U.S.; SD. HIV 1/2 3.0, SD. Korea Co., Ltd, Korea; and Determine HIV, Alere Medical Co., Matsudo, Japan. Testubg followed the national HIV testing algorithm.

Gonorrhea, *Chlamydia*, mycoplasma, and ureaplasma were tested for using PCR (Panamax Viral DNA/RNA, PANAGENE Inc., Daejeon, Korea). Syphilis was screened for using rapid plasma reagin (RPR carbon kit, Lorne Laboratories Ltd, UK). Positive specimens were confirmed using the *Treponema pallidum* haemagglutination assay (TPHA Microtiter kit, Lorne Laboratories Ltd, UK) at HHDV.

Data Management and Analysis

All interview answer sheets were reviewed by the investigators for any missing information. These sheets were stored in locked cabinets in HHDV. Data were entered using Epi-Data version 3.1 (EpiData Association, Odense, Denmark), and all statistical analyses were conducted using Stata version 14.0 (StataCorp, Station, TX, USA). Continuous variables were described using mean, median and range. Categorical variables were presented as proportions.

Conditional univariate and multivariate logistic regressions were used to assess risk factors for US NmNG urethritis. Given the small sample size, we used forward selection to add in variables one by one: those variables in univariate analysis giving p values lower than the others were included in a multivariate model. A log likelihood-ratio test was used to select the better fit model between the former (without the add-in variable) and the current (with the add-in variable) models. If the test gave $p<0.1$, the newly added variable was retained in the model. If any two variables were thought to be highly correlated with each other, only one was included in multivariate analysis.

References

1. Clinical and Laboratory Standards Institute. Performance standards for antimicrobial susceptibility testing; thirtieth informational supplement (M100–S30). Wayne (PA): The Institute; 2020.

2. Bolger AM, Lohse M, Usadel B. Trimmomatic: a flexible trimmer for Illumina sequence data. Bioinformatics. 2014;30:2114–20. PubMed https://doi.org/10.1093/bioinformatics/btu170

3. Bankevich A, Nurk S, Antipov D, Gurevich AA, Dvorkin M, Kulikov AS, et al. SPAdes: a new genome assembly algorithm and its applications to single-cell sequencing. J Comput Biol. 2012;19:455–77. PubMed https://doi.org/10.1089/cmb.2012.0021

4. Jolley KA, Bray JE, Maiden MCJ. Open-access bacterial population genomics: BIGSdb software, the PubMLST.org website and their applications. Wellcome Open Res. 2018;3:124. PubMed https://doi.org/10.12688/wellcomeopenres.14826.1

5. Retchless AC, Kretz CB, Chang H-Y, Bazan JA, Abrams AJ, Norris Turner A, et al. Expansion of a urethritis-associated *Neisseria meningitidis* clade in the United States with concurrent acquisition of N. gonorrhoeae alleles. BMC Genomics. 2018;19:176–76. PubMed https://doi.org/10.1186/s12864-018-4560-x

6. Brooks A, Lucidarme J, Campbell H, Campbell L, Fifer H, Gray S, et al. Detection of the United States *Neisseria meningitidis* urethritis clade in the United Kingdom, August and December 2019—emergence of multiple antibiotic resistance calls for vigilance. Euro Surveill. 2020;25:2000375. PubMed https://doi.org/10.2807/1560-7917.ES.2020.25.15.2000375

7. Hill V, Baele G. Bayesian estimation of past population dynamics in BEAST 1.10 using the skygrid coalescent model. Mol Biol Evol. 2019;36:2620–8. PubMed https://doi.org/10.1093/molbev/msz172

8. Croucher NJ, Page AJ, Connor TR, Delaney AJ, Keane JA, Bentley SD, et al. Rapid phylogenetic analysis of large samples of recombinant bacterial whole genome sequences using Gubbins. Nucleic Acids Res. 2015;43:e15. PubMed https://doi.org/10.1093/nar/gku1196

9. Nguyen L-T, Schmidt HA, von Haeseler A, Minh BQ. IQ-TREE: a fast and effective stochastic algorithm for estimating maximum-likelihood phylogenies. Mol Biol Evol. 2015;32:268–74. PubMed https://doi.org/10.1093/molbev/msu300

10. Rambaut A, Lam TT, Max Carvalho L, Pybus OG. Exploring the temporal structure of heterochronous sequences using TempEst (formerly Path-O-Gen). Virus Evol. 2016;2:vew007. PubMed https://doi.org/10.1093/ve/vew007

Appendix Table 1. Coinfection with other sexually transmitted infections among 19 cases of United States *Neisseria meningitidis* urethritis clade*

Symptoms	No STI coinfections, n = 12	STI coinfections, n = 7	p value†
Pyuria	0	2 (29)	0.12
Dysuria	10 (83)	3 (43)	0.13
Burning sensation during urination	1 (8)	1 (14)	1.0
Dripping	2 (17)	2 (29)	0.60
$\geq$1 symptom	10 (83)	6 (86)	1.0

*Values are no. (%). STI, sexually transmitted infection.
†Fisher exact test

Appendix Table 2. Genome typing of isolates from 19 cases of United States *Neisseria meningitidis* urethritis clade, Vietnam*

Isolate no.	Region	Year	Serogroup / genegroup	ST	PorA type	FetA VR	PorB type	FHbp peptide	NadA peptide	NHbA peptide	*gyrA*	*penA*	*rpoB*
20149	Ho Chi Minh	2019	NG/C	11	P1.5–1,10–8	F3–6	2–2	896	2	20	382†	316	8
20152	Dong Nai	2019	NG/C	11	P1.5–1,10–8	F3–6	2–2	896	(–)‡	20	4	316	8
20153	An Giang	2019	NG/C	11	P1.5–1,10–8	F3–6	2–2	896	(–)§	20	381†	316	8
20154	Tay Ninh	2020	NG/C	11	P1.5–1,10–8	F3–6	2–2	896	218†	20	4	316	8
20155	Ho Chi Minh	2019	NG/C	11	P1.5–1,10–8	F3–6	2–2	896	2	20	381†	316	8
20156	Ho Chi Minh	2019	NG/C	11	P1.5–1,10–8	F3–6	2–2	896	(–)‡	20	4	316	8
20157	Ho Chi Minh	2019	NG/C	11	P1.5–1,10–8	F3–6	2–2	896	2	20	381†	316	8
20158	Ho Chi Minh	2019	NG/C	11	P1.5–1,10–8	F3–6	2–2	896	2	20	381†	316	8
20161	Ho Chi Minh	2019	NG/C	11	P1.5–1,10–8	F3–6	2–408†	896	2	20	4	316	8
20162	Long An	2019	NG/C	11	P1.5–1,10–8	F3–6	2–39	896	2	20	4	316	8
20163	Ho Chi Minh	2019	NG/C	11	P1.5–1,10–1	F3–6	2–2	896	2	20	381†	316	8
20164	Ho Chi Minh	2019	NG/C	11	P1.5–1,10–8	F3–6	2–2	896	1	20	381†	316	8
20165	Ho Chi Minh	2019	NG/C	11	P1.5–1,10–8	F3–6	2–408†	896	2	20	4	316	8
20166	Ho Chi Minh	2020	NG/C	11	P1.5–1,10–8	F3–6	2–2	896	2	20	381†	316	8
20167	Can Tho	2019	NG/C	11	P1.5–1,Δ	F3–6	2–2	896	2	20	381†	316	8
20168	Ho Chi Minh	2019	NG/C	12881	P1.5–1,10–8	F1–26	2–2	896	2	20	4	316	8
20414	Ho Chi Minh	2020	NG/C	11	P1.5–1,10–8	Inc.	2–2	896	2	20	4	316	8
20415	Ho Chi Minh	2020	NG/C	11	P1.5–1,10–8	F3–6	2–2	896	2	20	4	316	8
20416	Ho Chi Minh	2020	NG/C	11	P1.5–1,10–8	Inc.	2–2	896	2	20	4	316	8

*Inc., incomplete coding; NG, nongroupable; ST, sequence type.
†New allele.
‡Contains an inserted element
§Internal stop codon.

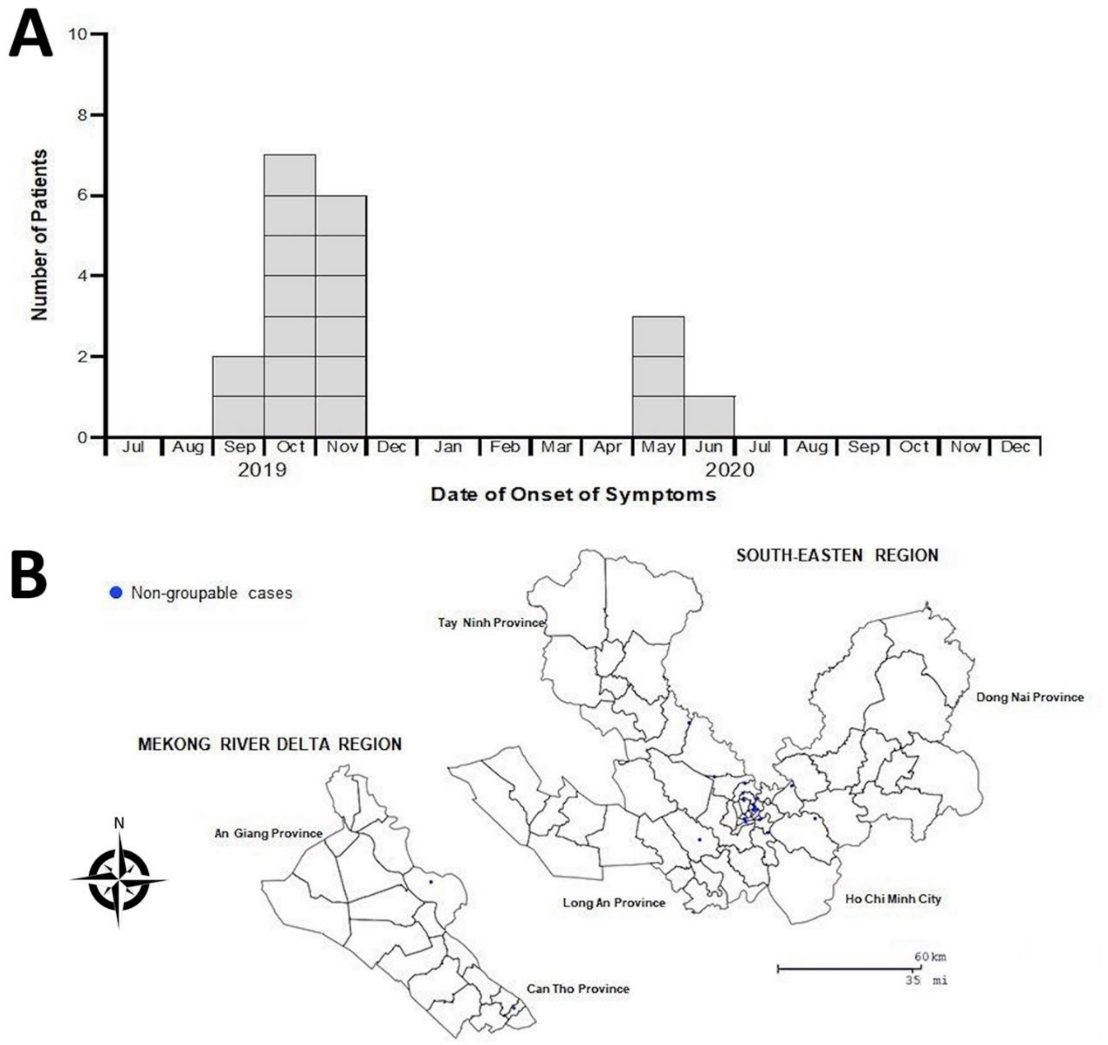

Appendix Figure 1. Number of cases per month and locations in an outbreak of sexually transmitted nongroupable *Neisseria meningitidis*–associated urethritis, Vietnam. A) Number of cases detected per month during 2019–2020. B) Locations of patient residences in Vietnam.

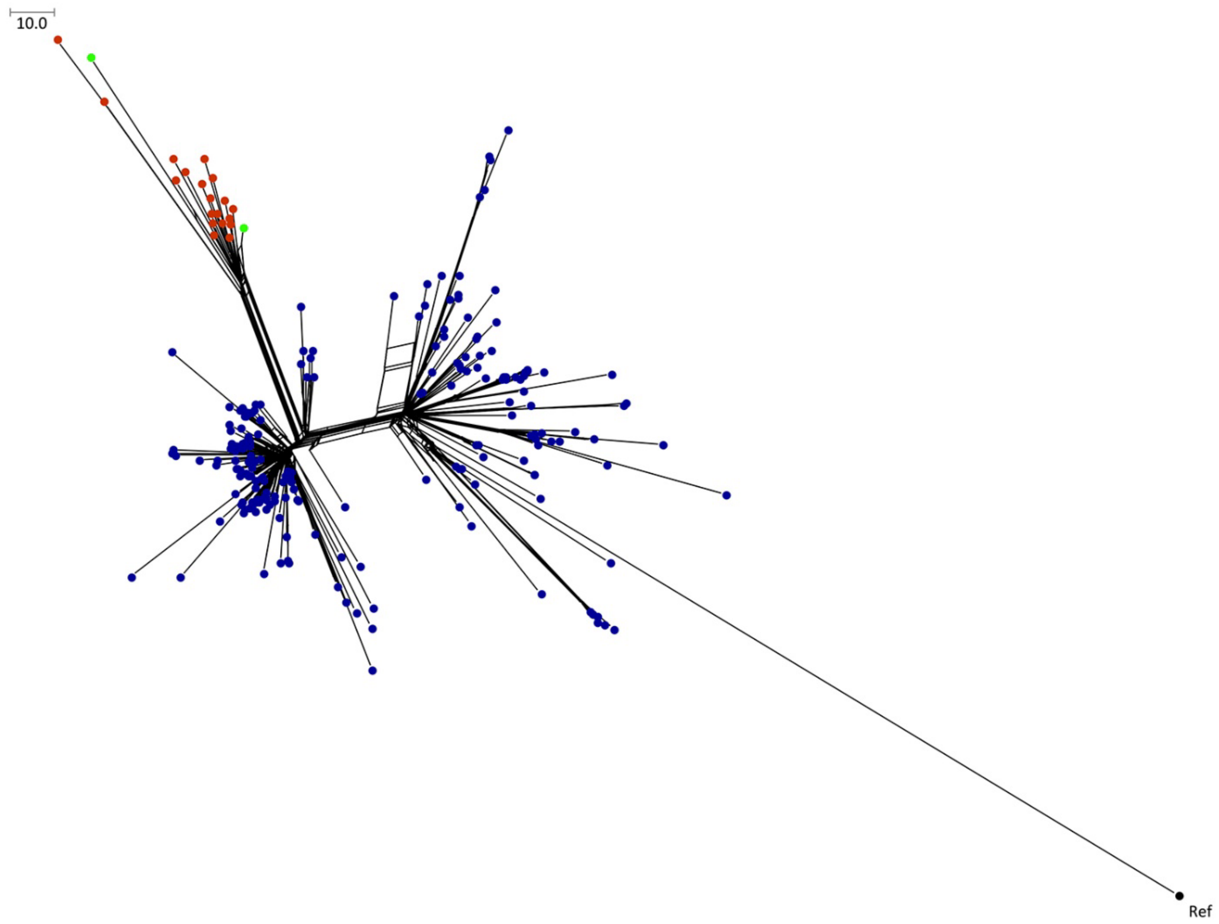

Appendix Figure 2. Phylogenic tree of isolates used to study an outbreak of sexually transmitted nongroupable *Neisseria meningitidis*–associated urethritis, Vietnam. The phylogenetic tree displayed the isolates in Vietnam (red dots) and the United Kingdom (green dots) forming a monophyletic clade with those from Ohio (blue dots), one of two clades of US NmNG urethritis. Scale bar indicates nucleotide substitutions per site.

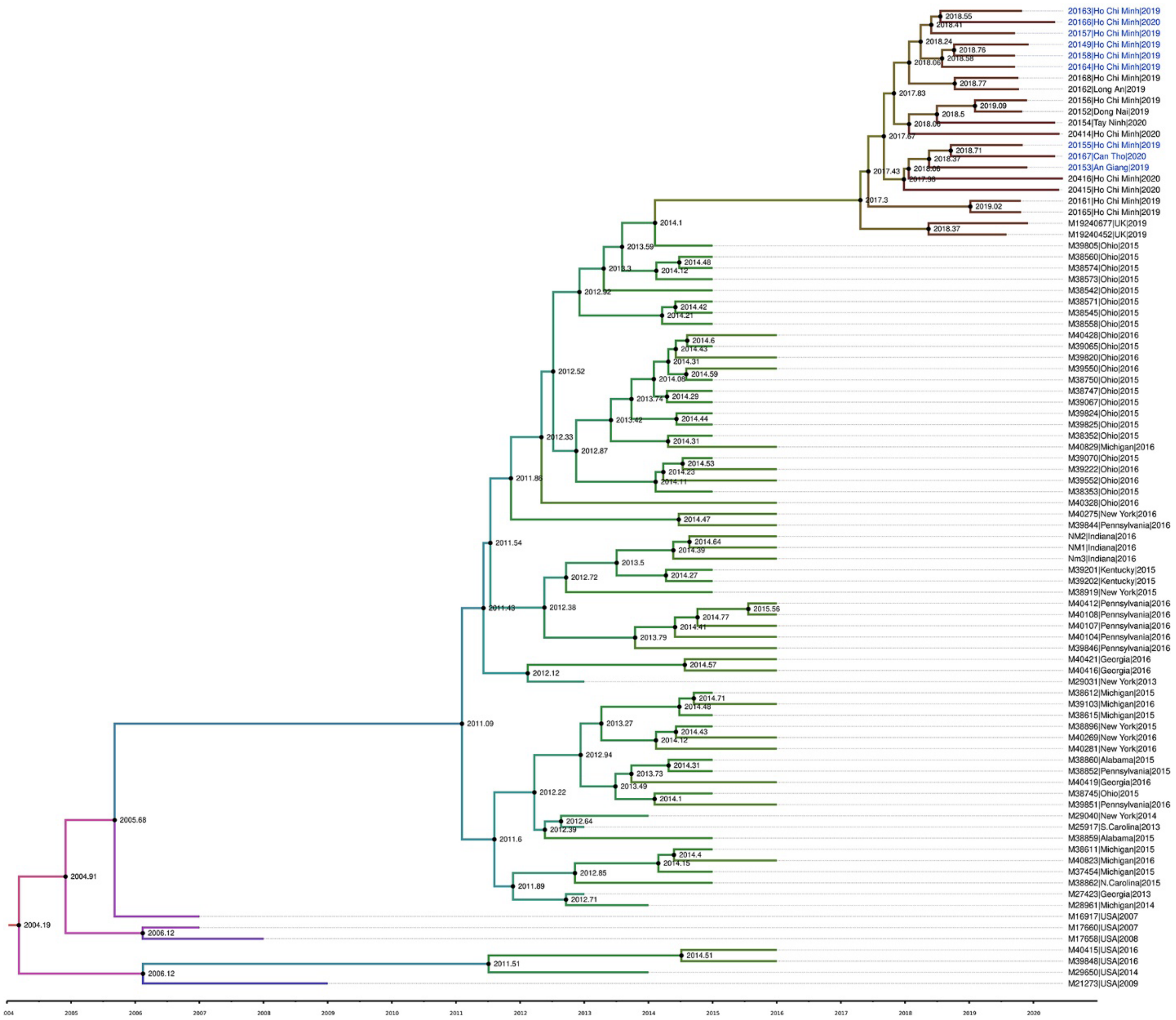

Appendix Figure 3. Evolutionary plylogenetic tree of isolates from an outbreak of sexually transmitted nongroupable *Neisseria meningitidis*–associated urethritis, Vietnam. Phylogenetic tree was constructed using Baysian Skygrid model, performing with BEAST/BEAGLE v1.10.4 (https://beast.community/beagle), and displaying with FigTree v1.4.4 (http://tree.bio.ed.ac.uk/software/Figtree). Isolates from this outbreak are detailed in Figure 1. Scale bar indicates the time of evolutionary history.
